# Prevalence of GB virus type C in urban Americans infected with human immunodeficiency virus type 1

**DOI:** 10.1186/1742-4690-2-38

**Published:** 2005-05-31

**Authors:** Stephen M Smith, Michael J Donio, Mahender Singh, James P Fallon, Lavanya Jitendranath, Natalia Chkrebtii, Jihad Slim, Diana Finkel, George Perez

**Affiliations:** 1 Saint Michael's Medical Center, Newark New Jersey 07102, USA; 2 The New Jersey Medical School, Newark New Jersey 07102, USA

## Abstract

GBV-C virus infection has been linked to improved clinical outcome in HIV-1 co-infected individuals. The epidemiology of GBV-C has, thus far, been limited to the gay male, HIV^+ ^population. Here we describe the prevalence of antibodies against GBV-C envelope glycoprotein E2 and GBV-C viremia in an HIV^+ ^inner city population. This study group is predominantly African-American; 41% of the participants are women. The major risk factor for HIV infection is intravenous drug use. Overall, 56% of the study population had evidence of current or past infection with GBV-C. GBV-C exposure was not associated with hepatitis C virus infection. The group of participants, who had GBV-C viremia and anti-E2 antibodies, had high percentage of patients with an undetectable HIV-1 viral load. These data provide increased insight into the prevalence of GBV-C co-infection in the HIV epidemic in this understudied population.

## Background

In 1995, several groups independently reported the discovery of two new viruses, which were termed GB virus type C (GBV-C) and hepatitis G virus, respectively (review in [1]). Subsequently, these viruses were found to be two strains of a novel RNA virus belonging to the *Flaviviridae *family. GBV-C (the designation used in this paper) is distantly related to hepatitis C virus (HCV) with which it shares approximately 30% amino acid homology. While HCV replicates primarily in hepatocytes, GBV-C replicates in both T- (CD4^+ ^and CD8^+^) and B-lymphocytes. GBV-C is not known to cause disease in humans, but can establish chronic infection in which virus may be present in the blood. After years of infection, infected individuals may spontaneously clear GBV-C [1], although the reasons for this phenomenon are not known. In most cases, clearance of GBV-C is associated with seroconversion to the viral envelope glycoprotein, E2. Paradoxically, viremia may also persist despite the presence of anti-E2 antibodies, and clearance may occur in the absence of seroconversion. GBV-C may be transmitted through several routes, including sexual contact, exposure to contaminated blood and vertical transmission. To date, the epidemiology of GBV-C is incompletely understood.

Of interest, GBV-C infection appears to alter the course of human immunodeficiency virus type 1 (HIV-1) infection. Following an initial report in 1998 [2], several studies have shown that individuals, who are co-infected with GBV-C and HIV-1, have lower levels of HIV-1 viremia and higher CD4^+ ^T cell counts than those infected with HIV-1 alone [3-8]. However, other studies have not supported this association [9-13]. A recent report failed to find evidence that active GBV-C co-infection improved survival 12 to 18 months after HIV-1 seroconversion [6]. Survival rates in persons with persistent GBV-C viremia were, however, significantly better 5 to 6 years after HIV-1 infection.

GBV-C prevalence is known to be significantly higher in HIV-1 seropositive individuals (>75%) [3,5,6,13] compared with healthy blood donors (10–20%) [14]. In most cases, this observation is based on evaluation of patient groups comprised primarily of men, who have sex with men (MSM). The epidemiology of GBV-C among HIV-1 seropositive, inner city residents, whose risk factors, ethnicity and gender are distinct, is not known. In the present study, we evaluated the prevalence of GBV-C infection in a population consisting primarily of HIV-infected, urban African-Americans.

## Methods

### Study Population

The study population consisted of 353 HIV-1-infected patients who regularly attended a large urban HIV-1 clinic. The patients were recruited over a 3-month period between February and April 2004. The study was approved by the institutional review board of Saint Michael's Medical Center and informed consent was obtained from all participants prior to sample collection. Blood samples were obtained for analysis of GBV-C RNA and anti-E2 antibodies, and for measurement of HIV-1 plasma RNA levels, CD4^+ ^T-cell counts and HCV serology. Treatment was independently determined by the treating physician.

### Laboratory Assays

Studies for HIV RNA levels, HIV antibodies, and HCV antibodies were performed by commercial laboratories.

### RT-PCR for GBV-C RNA

Total RNA was extracted from 100 μl of serum using an RNAeasy Mini Kit (Qiagen, Valencia, CA). Twenty-five percent of the isolated RNA was used for reverse transcription (RT) and first round PCR. RT-PCR was performed in a single tube using the AccessQuick RT-PCR System (Promega, Madison, WI). Both first- and second-round PCR were carried out using primers that hybridize to 5' non-translated regions of an infectious GBV-C clone (GenBank accession no. AF121950, nt 54 to 389). Primers for the first-round RT-PCR were GBVF1 5'-CCGACGCCTATCTAAGTA GACGC and GBVR1 5'-TCAACTCGCCGGATAAACCTATTGG. Primers for the second-round PCR were GBVF2 5'-GTGACAGGGTTGGTAGG and GBVR2 5'-GACATTGAAGGGCGACGTGG. PCR products were detected on 1.5% agarose gels containing 0.5 μg/ml ethidium bromide. The expected band sizes were 336 and 231 bp for the first- and second-round PCR, respectively. Known GBV-C positive serum (generously provided by Dr. J. Stapleton, University of Iowa, Iowa City, IA) and negative (saline) controls were included in each assay. Samples yielding ambiguous PCR results were re-tested using freshly extracted RNA from the original sera. A reaction was considered positive if either the first- or second-round PCR produced a band of the expected size. The assay was validated using *in vitro *transcribed GBV-C RNA together with positive and negative control samples.

### Detection of Antibodies against GBV-C glycoprotein E2

Antibodies against GBV-C envelope glycoprotein E2 were detected using the μPlate anti-HGenv ELISA test (Roche Diagnostics, Indianapolis, IN) according to the manufacturer's protocol, which is summarized below. A 1:20 dilution of each serum sample was added to an incubation solution containing HGV-E2 antigen-bound, biotinylated anti-E2 antibodies. This solution was then added to a streptavidin-coated microwell plate. After the plate was washed once with the wash-solution, POD-labeled, anti-human Fcγ antibodies were added to the plate, followed by ABTS^® ^substrate solution. Absorbance was read at 405 nm. A sample was considered positive if the A_405 _≥ the cut-off value calculated according to the manufacturer's protocol (0.2 times the sum of the positive and negative controls). Samples falling within +/-15% of the cut-off value were repeated using freshly diluted sera.

### Statistical Analyses

A chi-square or Fisher's exact test was used to analyze categorical variables. The group means were compared by either the Student's t-test, Mann-Whitney U test or Wilcoxon rank sum test. p values <0.05 were considered to indicate statistical significance and all reported p values were two-sided.

## Results

### GBV-C prevalence

Of the 353 subjects studied here, 208 (59%) were men and 145 (41%) were women. The mean age within the cohort was 46.4 years, and the mean CD4^+ ^T-cell count was 416 cells/mm^3^. Plasma from each patient was tested for the presence of GBV-C RNA and anti-glycoprotein E2 antibodies. GBV-C RNA was detected in 23.2% (82/353) of subjects. Among those testing positive for GBV-C RNA, 13 (16%) were also found to be positive for anti-E2 antibodies. Within the study population, 32.3% (114/353) tested positive for anti-E2 antibodies alone, while the remaining subjects (157/353) tested negative for both GBV-C RNA and anti-E2 antibodies. Overall, 56% of the study population had evidence of GBV-C exposure, defined as a positive result for either GBV-C RNA or anti-E2 antibodies.

Among the study subjects, the main HIV-1 risk factors were intravenous drug use (IDU) (53.8%) and heterosexual contact with a person who used intravenous drugs (32.6%) (Table 1). The majority of the enrolled population (71.4%) was African-American. There was no significant difference in GBV-C status between sex, race or HIV-1 risk factor (Table 2).

**Table 1:** HIV-1 risk factors according to GBV-C status.

	GBV-C RNA positive	Anti- E2 antibody positive alone	Negative for GBV-C RNA and Anti-E2 antibody	Total
Hemophilia n (%)*	1 (11%)	2 (22%)	6 (67%)	9 (100%)
Heterosexual	25 (22%)	38 (33%)	52 (45%)	115 (100%)
Heterosexual/IVDU	10 (24%)	10 (24%)	12 (52%)	42 (100%)
IVDU	35 (22%)	58 (37%)	65 (41%)	158 (100%)
MSM	8 (35%)	2 (9%)	13 (66%)	23 (100%)
Transfusion	2 (20%)	3 (30%)	5 (50%)	10 (100%)
Other	1 (17%)	1 (17%)	4 (67%)	6 (100%)

*- percentage by risk factor

**Table 2:** Demographics of the study population.

	GBV-C RNA positive	Anti- E2 positive	Unexposed to GBV-C	All subjects
Total	82	114	157	353
Age*	44.7 ± 7.9	47.0 ± 8.9	46.8 ± 9.5	46.4 ± 9.0
				
Sex n (%)				
Male	52 (63%)	65 (57%)	91 (58%)	208 (59%)
Female	30 (37%)	49 (43%)	66 (42%)	145 (41%)
				
Race n (%)				
Black	65 (79%)	83 (73%)	104 (66%)	252 (71%)
Hispanic	6 (7%)	18 (16%)	31 (20%)	55 (16%)
Caucasian	11 (13%)	13 (11%)	22 (14%)	46 (13%)

* -average ± S.D.

Although GBV-C is thought to be transmitted by similar routes as HCV, HCV antibody status was not strongly associated with GBV-C exposure (Table 3). A total of 163 subjects were positive for HCV antibodies. Among those, 61% (99/163) were also positive for either GBV-C viremia or anti-E2 antibody, while 51% (97/190) of the HCV antibody negative population had evidence of GBV-C exposure.

**Table 3:** The presence of hepatitis C virus antibody according to GBV-C status.

HCV Antibody Status	GBV- C RNA positive n (%)	Anti-E2 antibody positive n (%)	Negative for GBV-C RNA and Anti-E2 antibody n (%)
			
HCV (+)	39 (48%)	60 (53%)	64 (41%)
HCV (-)	43 (52%)	54 (47%)	93 (59%)
Total	82 (100%)	114 (100%)	157 (100%)

### Implications of GBV-C viremia

Although this study represents a cross-sectional analysis, we evaluated the relationship between GBV-C infection status and HIV-1 viremia or CD4^+ ^T cell count. GBV-C exposure was associated with a larger percentage of patients with CD4^+ ^T cell counts >350 cells/mm^3 ^(p < 0.05, Table 4). In addition, 46.3% of patients with GBV-C viremia had plasma HIV-1 RNA levels <500 copies/ml, compared with 36.9% of patients who tested negative for GBV-C RNA and anti-E2 antibodies. This trend, while suggestive of an effect of GBV-C co-infection on HIV-1, was not statistically significant. However, a separate analysis of the 13 patients who tested positive for both GBV-C RNA and anti-E2 antibodies revealed a significant association between GBV-C infection and low plasma HIV-1 RNA levels (p < 0.05, Table 5). Overall, for the entire cohort, GBV-C status had no effect on the mean plasma HIV-1 RNA levels or the mean CD4^+ ^T-cell count.

**Table 4:** Patient stratification by CD4^+ ^T-cell count, according GBV-C exposure status.

	Exposed to GBV-C n (%)	Negative for GBV-C RNA and Anti-E2 antibody n (%)
CD4^+ ^T-cell count		
≤ 350 cells/mm^3^	81 (41%)	82 (52%)
>350 cells/mm^3^	115 (59%) †	75 (48%)
Total	196 (100%)	157 (100%)

† The percentage (115/196; 59%) of GBV-C exposed subjects with a CD4^+ ^T-cell count >350 cells/mm^3 ^was higher than that (75/157; 48%) of those unexposed to GBV-C (p < 0.05; Chi-square test).

**Table 5:** Plasma HIV-1 RNA levels according to GBV-C status.

HIV RNA copies/mL	GBV-C RNA positive/ anti-E2 antibody positive n (%)	GBV-C RNA positive n (%)	Anti-E2 antibody positive n (%)	Negative for GBV-C RNA and Anti-E2 antibody n (%)
				
≤ 500	10 (77%) ^†^	28 (41%)	46 (40%)	58 (37%)
>500	3 (23%)	41 (59%)	68 (60%)	99 (63%)
Total	13 (100%)	69 (100%)	114 (100%)	157 (100%)

p < 0.05 when comparing RNA+/E2+ group to other GBV-C status groups.

† Comparison among the four groups were made using the Chi-square test.

## Conclusion

This report is the first to describe the prevalence of GBV-C in an inner city population comprised predominantly of HIV-1-infected African-Americans. Previous studies, which focused on HIV-seropositive male homosexuals, found evidence of prior or current GBV-C infection in 74 to 85% of subjects [3,5,6,13]. In contrast, only 56% of the population studied here tested positive for GBV-C RNA and/or anti-E2 antibodies. Analysis of HIV-1 risk factors did not reveal a significant correlation between specific high-risk behavior for HIV-1 and GBV-C exposure.

Our findings comprise data from the largest such cohort studied to date. Within our study population, we found a 61% rate of GBV-C exposure among 163 HCV antibody positive patients; a rate not significantly different from that of HCV antibody negative patients (51%). Taken together with published findings, these data suggest that GBV-C may be transmitted more efficiently by male homosexual contact than by either intravenous drug use or heterosexual contact. Alternatively, infection with GBV-C may have occurred at a higher rate in our population, but either GBV-C viremia or anti-E2 antibody responses may have waned over time in some patients. Pre-exposure to GBV-C may have rendered individuals within the population immune to secondary infection with this flavivirus. Within this cohort, we also studied a large population of HIV-infected women (n = 145) and found a similar rate of GBV-C exposure (55%) to that found in men (57%).

Although the effect of GBV-C on HIV-1 infection remains controversial, most studies have shown higher CD4^+ ^cell counts and lower HIV-1 viral loads in GBV-C viremic patients. Our study suggests a similar effect among a population with distinct characteristics including ethnicity, transmission profiles and gender than those previously reported. In our cohort, 77% of patients who tested positive for both GBV-C RNA and anti-E2 antibodies had plasma HIV-1 RNA levels <500 copies/ml. While these data represent a small number of patients, the percentage with low HIV-1 viral load was statistically higher among these patients when compared to those in the other groups. The co-existence of GBV-C viremia and anti-E2 antibody may be a marker of long-term GBV-C infection, which has recently been shown to correlate with a better outcome in HIV-1-infected individuals [6].

Our study was limited in several aspects, including a lack of longitudinal follow-up. In addition, we were unable to compile adequate data on the duration of HIV-1 infection and the possible impact of antiretroviral therapy in these patients. The latter restriction may be balanced by the fact that nearly all HIV-infected patients in this large cohort received antiretroviral therapy under a relatively consistent standard practiced within a single clinical environment.

Further longitudinal studies will be necessary in HIV-1-infected patients to clarify the potential effects of GBV-C co-infection. Our data support the hypothesis that GBV-C viremic patients with HIV-1 respond better to therapy, which has been suggested by another study [15]. This possibility needs to be tested prospectively. Our data also suggest that the behaviors associated with HIV-1 transmission in the inner city are less associated with GBV-C exposure than in other high-risk settings.
